# Blood coagulation factors and risk of venous thromboembolism: a population-based study

**DOI:** 10.1186/s40001-025-03799-3

**Published:** 2026-01-11

**Authors:** Anna Erhard, Dennis Freuer, Annette Peters, Margit Heier, Christa Meisinger, Jakob Linseisen

**Affiliations:** 1 Epidemiology, Medical Faculty, University of Augsburg, Stenglinstr. 2, 86156 Augsburg, Germany; 2 Institute of Epidemiology, Helmholtz Zentrum München – German Research Center for Environmental Health (GmbH), Neuherberg, Germany; 3 Chair of Epidemiology, Institute for Medical Information Processing, Biometry and Epidemiology, Medical Faculty, Ludwig-Maximilians-Universität München, Munich, Germany; 4 German Centre for Cardiovascular Research (DZHK), Partner Site Munich Heart Alliance (MHA), Munich, Germany; 5 KORA Study Centre, University Hospital of Augsburg, Augsburg, Germany

**Keywords:** Prevalence, Venous thromboembolism, Population-based study, KORA-Fit, Blood coagulation factors

## Abstract

**Background:**

The prevalence of venous thromboembolism (VTE) and the impact of blood coagulation factors on the occurrence of VTE in the general population have been rarely studied.

**Methods:**

In the KORA-Fit (S4) study with *n* = 805 participants (53% females) with a mean age of 62.4 (SD 5.7) years, the prevalence of physician-diagnosed VTE was assessed during a face-to-face interview. Plasma concentrations of antithrombin, fibrinogen, factor VIII, d-dimer, protein C, and protein S activity were analyzed; additionally, aPTT and prothrombin time were assessed as screening tests. The associations between coagulation factors and VTE were analyzed using multivariable logistic regression models. Non-linear associations were explored using restricted cubic splines.

**Results:**

The self-reported prevalence of VTE was 3.2%. The blood coagulation factors examined did not differ between individuals with and without VTE, except for factor VIII. In multivariable analyses, protein C (adjusted *p* = 0.008), protein S (adjusted *p* = 0.008), and aPTT (adjusted *p* = 0.016) were non-linearly inversely associated with VTE.

**Conclusions:**

Our findings in a middle-aged and elderly population demonstrate significant associations between specific blood coagulation factors and the prevalence of VTE. However, these hypothesis-generating findings must be confirmed in prospective cohort studies. Also, research on the complex interactions between coagulation factors and patient-specific risk factors in the pathogenesis of VTE is warranted.

**Supplementary Information:**

The online version contains supplementary material available at 10.1186/s40001-025-03799-3.

## Background

Venous thromboembolism (VTE) includes deep vein thrombosis (DVT) of the leg or pelvis, and its complication, pulmonary embolism (PE). As one of the manifestation forms of cardiovascular disease, VTE has a big impact on the quality of life of the affected patients; still, it is responsible for 60,000 to 100,000 deaths worldwide annually. Depending on the data source, the incidence of first-time VTE varies between 0.71 and 1.43 per 1000 person-years, and this rate increases with age [1–6].

DVT is the clinical consequence of clot formation inside a blood vessel. The most common risk factors for DVT are surgery or trauma, any kind of immobilization, oncological disease, pregnancy, heart failure, chronic venous insufficiency, or a DVT in the past [6]. Also, lifestyle factors such as obesity (BMI > 30 kg/m^2^), smoking, or use of estrogen or contraceptives are risk factors for DVT [7]. The most widespread hereditary factors, which increase the risk of DVT are Activated Protein C (APC)-Resistance/Factor V-Leiden mutation, prothrombin mutation, or protein C, protein S, or antithrombin (AT) deficiency [8].

PE is a heavy and underdiagnosed complication which occurs in 50% of DVT patients. Thereby, one of the pulmonary arteries is occluded by an embolus, which—in more than 90% of the cases—originated from the venous system of lower extremities or pelvic veins [7]. In about 45% of the cases, the diagnosis of PE is made ante mortem [9]. Despite preventive measures taken routinely during hospital stays, PE is still the leading comorbidity and cause of death in postsurgical and pregnant patients in hospitals, as well as shortly after discharge [10].

Hemostasis is a complex physiological process involving interactions between platelets, coagulation factors, and the vessel wall to prevent blood loss and maintain vascular integrity [11]. Blood coagulation factors have a direct impact on the balance between two extreme conditions—thromboembolism and bleeding [7]. However, research on blood coagulation factors and their impact as possible risk factors for VTE in the adult general population is still scarce. The exploration of this possible connection can be a relevant step for improving prevention of VTE. Therefore, in the present study, the prevalence of VTE and the association between various coagulation factors and VTE were investigated based on data of a large sample from the general population.

## Methods

### Study participants

The data for this analysis came from the KORA project, which stands for Cooperative Health Research in the Region of Augsburg. The KORA project itself was established as a continuation of the WHO-MONICA (monitoring trends and determinants in cardiovascular disease) project in 1996, which had the purpose of researching cardiovascular morbidity and mortality worldwide [12, 13]. Altogether four cross-sectional baseline surveys (S1, S2, S3, and S4) were conducted between 1984 and 2001. In the years 2018 and 2019, the follow-up study KORA-Fit followed, to which all KORA participants born between 1945 and 1964 were invited for a re-examination. Finally, 3059 eligible subjects participated in the KORA-Fit Study, i.e., 64.4% of all eligible persons [14]. Out of these, 805 participants (378 male and 427 female) originally recruited in KORA S4, and in 2018/19 participants of KORA-Fit provided citrate plasma samples for the measurement of coagulation factors (see Figure S1, Supplementary material). The study was approved by the Ethics Committee of the Bavarian Chamber of Physicians and conducted according to the data protection requirements (Ethics-No. 17040). All subjects gave written informed consent to participate in the study. The study was conducted in accordance with the Declaration of Helsinki.

### Data collection

Sociodemographic data (e.g., education level) were collected by trained medical staff during a standardized interview in the study center. During the interview, information on common cardiovascular risk factors (e.g., smoking habits, physical activity) and comorbidities, such as the history of cancer, was collected. It was also registered if a participant had received a physician-based diagnosis of DVT and/or PE in the past. During the physical examination, anthropometric data and blood pressure measurements were assessed following a standardized protocol [15]. The level of education was assessed in school years. Subjects were considered physically active, if they declared leisure time physical activity for at least one hour per week during winter and summer.

### Laboratory measurements

In addition to the interview and physical examinations, venous blood samples were collected after overnight fasting. The standard clinical parameters measured included serum total cholesterol, HDL cholesterol, triacylglyceride concentrations, and hs-CRP. From all 805 participants citrate plasma samples were collected, and Table S1 (see Supplementary material) shows the analyzed blood coagulation factors and methods used for their analysis. The levels of the most common screening parameters such as aPTT and prothrombin time were determined, which display the state of the coagulation system. Also, some clinically relevant coagulation factors and proteins were tested, namely AT, fibrinogen, factor VIII, d-dimer, protein C, and protein S. The coagulation factors were analyzed at the clinical laboratory of the University Hospital Augsburg. Standard clinical laboratory parameters were analyzed immediately at the clinical laboratory of the University Hospital Großhadern (Ludwig-Maximilians Universität, München).

### Statistical analyses

Regarding the distributions, continuous data were presented as median as well as interquartile range (25th and 75th percentile) and group differences were assessed using the Mann–Whitney *U* test. For categorical data, which were presented as absolute and relative frequencies, Fisher’s exact test was performed.

To investigate the relationship between coagulation factors (independent variables) and VTE, odds ratios (OR) and 95% confidence intervals (95% CI) were calculated in multivariable binary logistic regression models. When examining the relationship between prothrombin time and aPTT and the outcome, study participants using anticoagulant agents (*n* = 28) were excluded. All models were adjusted for age, sex, BMI, physical activity, smoking status, prevalence of diabetes mellitus, cancer, estimated glomerular filtration rate (eGFR), and hs-CRP. Absence of multicollinearity was verified by calculating the variance inflation factor (VIF). Linearity was tested and ensured using restricted cubic splines. Specifically, for each continuous independent variable in each model, the optimal number of knots between three and five was determined based on Akaike’s Information Criterion (AIC) and tested for a non-linear association against the linear model using the likelihood ratio test (see Table S2, Supplementary material). P-values from tests for non-linear associations of continuous independent variables with the logits of the respective outcomes are presented in Table S3 (see Supplementary material).

As the few missing values in the blood coagulation factors (less than 6% per variable) could be assumed completely at random due to technical issues, a multivariate imputation by chained equations (MICE) was performed creating 5 imputed datasets for subsequent regression analyses.

To account for multiple testing regarding 8 exposures, a Bonferroni corrected *p*-value < 0.05 was defined as statistically significant. Except for the regression models, which were conducted in R (version: 3.6.0), the statistical analyses were performed in SPSS (version 27.0.1.0) for MacOS.

## Results

Among all 805 KORA-Fit study participants with a median age of 63 (58; 67), 53% were females (*n* = 427), and 3.2% (*n* = 26) were diagnosed with VTE in the past (Table 1). In comparison to the no-VTE group, participants with prevalent VTE were older, less educated, had higher BMI, and higher serum creatinine levels, while eGFR levels were lower.

**Table 1:** Characteristics of the study participants (*n* = 805), stratified by sex and by the diagnosis of venous thromboembolism (VTE)

Characteristic	Total (*n* = 805)	Male (*n* = 378)	Female (*n* = 427)	*p*-value	no VTE (*n* = 779)	VTE (*n* = 26)	*p*-value
Age [years]	63 (58; 67)	62 (57; 67)	63 (58; 67)	0.988	63 (57; 67)	66 (61.5; 67.75)	0.044
Education [years]	11 (10; 13)	12 (10; 13)	11 (10; 13)	< 0.001	11 (10; 13)	10 (10; 12.8)	0.044
BMI [kg/m^2^]	27.6 (24.4; 31.1)	28.2 (25.49; 31.3)	26.8 (23.5; 30.5)	< 0.001	27.4 (24.3; 31.1)	28.5 (26.6; 32.9)	0.036
eGFR [mL/mil]	85.0 (75.4; 93.3)	86.5 (75.5; 94.5)	83.4 (74.9; 92.9)	0.053	85.4 (75.8; 93.6)	75.7 (64.8; 84.9)	< 0.001
Creatinine [mg/dL]	0.8 (0.7; 1.0)	0.9 (0.8; 1.0)	0.8 (0.7; 0.8)	< 0.001	0.8 (0.7; 1.0)	0.9 (0.8; 1.1)	0.008
hs-CRP [mg/L]	1 (1; 3)	1 (1; 3)	2 (1; 3)	0.062	1 (1; 3)	1.5 (1; 3)	0.996
Sex							0.752
Male	378 (0.47)	–	–	–	365 (0.469)	13 (0.500)	
Female	427 (0.53)	–	–	–	414 (0.531)	13 (0.500)	
Physical activity				0.506			0.281
Active	565 (0.702)	261 (0.69)	304 (0.712)		544 (0.698)	21 (0.808)	
Not active	240 (0.298)	117 (0.31)	123 (0.288)		235 (0.302)	5 (0.192)	
Smoking				< 0.001			0.389
Smoker	110 (0.137)	56 (0.148)	54 (0.126)		109 (0.14)	1 (0.038)	
Non-smoker	355 (0.441)	192 (0.508)	163 (0.382)		342 (0.439)	13 (0.5)	
Never-smoker	340 (0.422)	130 (0.344)	210 (0.492)		328 (0.421)	12 (0.462)	
Diabetes				0.375			0.262
Yes	69 (0.086)	36 (0.095)	33 (0.078)		65 (0.084)	4 (0.16)	
No	734 (0.914)	342 (0.905)	392 (0.922)		713 (0.916)	21 (0.84)	
Cancer				0.478			0.758
Yes	92 (0.114)	40 (0.106)	52 (0.122)		90 (0.116)	2 (0.077)	
No	713 (0.886)	338 (0.894)	375 (0.878)		689 (0.884)	24 (0.923)	
Anticoagulant use				0.001			< 0.001
Yes	28 (0.035)	22 (0.058)	6 (0.014)		20 (0.026)	8 (0.308)	
No	776 (0.965)	356 (0.942)	420 (0.986)		758 (0.974)	18 (0.692)	

Continuous variables were given as median and 25th and 75th percentiles. Categorical variables were given as absolute and relative numbers. Group differences were assessed using the Mann–Whitney *U* test for continuous and Fisher’s exact test for categorical variables

The distributions of all blood coagulation factors were within the physiological range (Table 2), except for d-dimers, which showed higher levels in both groups: 488.5 (310.5; 803.8) μg/L in the VTE group versus 405 (306; 553) μg/L in the no-VTE group (*p* = 0.203). In addition, participants with VTE showed higher levels of factor VIII as compared to the no-VTE group (Table 2).

**Table 2:** Concentrations of plasma coagulation factors for the total sample and by diagnosis of VTE in the past

Characteristic	Total (*n* = 805)	no VTE (*n* = 779)	VTE (*n* = 26)	*p*-value
Prothrombin time [%]^a^	108.8 (102.2; 114.5)	108.8 (102.2; 114.5)	106.8 (103.5; 117.1)	0.779
aPTT [sec.]^a,b^	30.7 (28.7; 32.9)	30.7 (28.7; 32.9)	29.9 (26.5; 31.8)	0.081
Antithrombin [%]	102.5 (95.5; 109.1)	102.7 (95.5; 109.2)	99.8 (95.0; 107.5)	0.651
Fibrinogen [mg/dL]	296.1 (261.5; 336.4)	295.8 (261.6; 335.8)	308.2 (254.9; 367.4)	0.246
D-dimer [µg/L]	405 (306; 556)	405 (306.0; 553.0)	488.5 (310.5; 803.8)	0.203
Protein C [%]	123.4 (111.4; 138.4)	123.5 (111.5; 138.5)	119.3 (95.8; 137.0)	0.175
Protein S [%]	126 (105.1; 146.5)	126.1 (105.5; 146.7)	109.2 (87.7; 142.3)	0.107
Factor VIII [%]	120.7 (97.6; 142.8)	120.3 (97.3; 142.8)	140.7 (121.0; 171.4)	0.007

Coagulation factor concentrations were given as median and 25th and 75th percentiles; differences between groups were tested using the Mann–Whitney *U* test

^a^Twenty-eight participants with anticoagulation therapy excluded

^b^Activated partial thromboplastin time

**Fig. 1:**
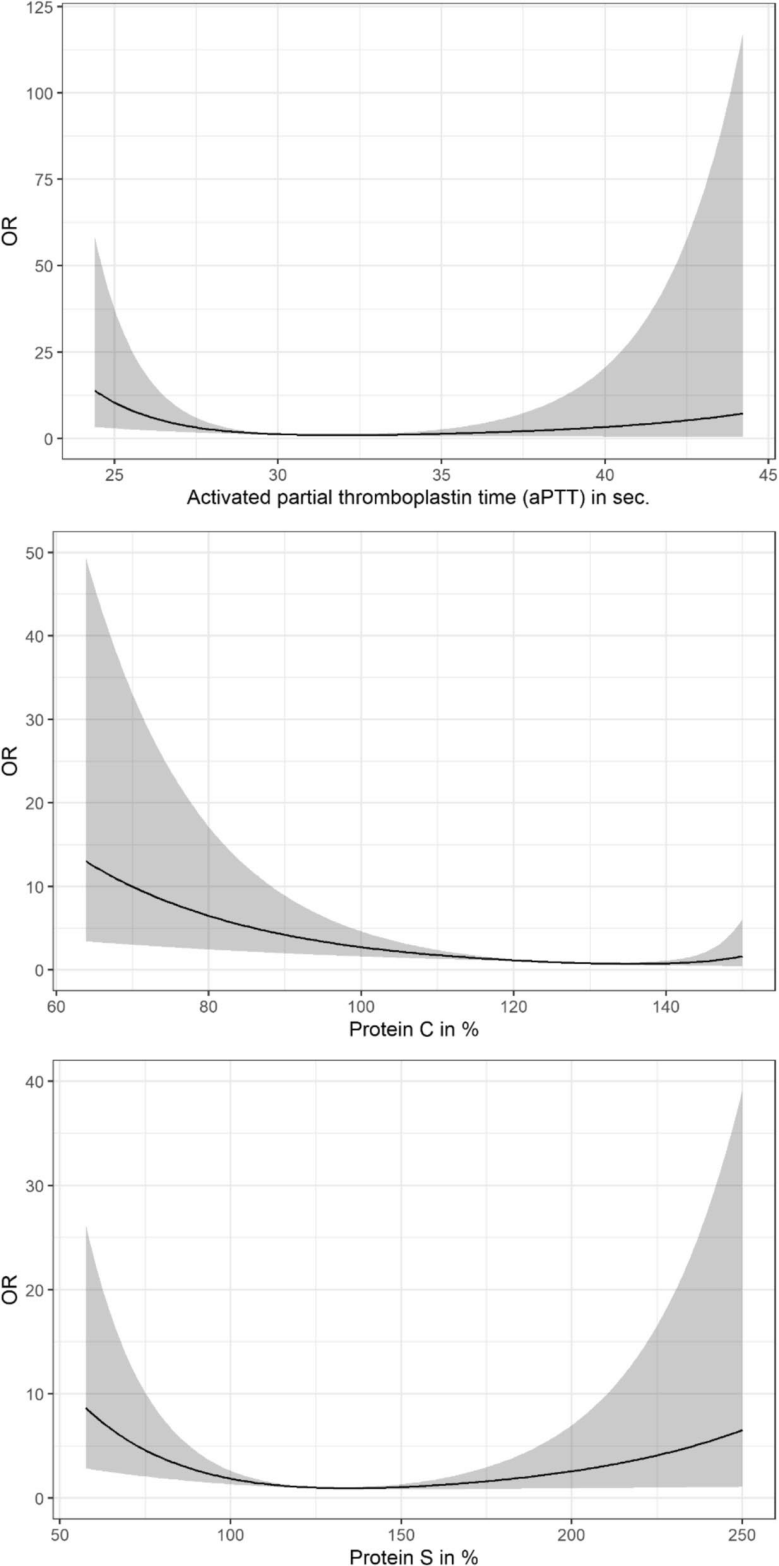
Non-linear associations of plasma aPTT, protein C, and protein S with VTE. The associations on the odds ratio (OR) scale and the respective 95% confidence bands derived from multivariable logistic regressions using restricted cubic splines with 3 knots (10%, 50%, and 90% quantiles). The Bonferroni-adjusted p-values for overall associations were *p* = 0.016 (aPTT), *p* = 0.008 (protein C), and *p* = 0.008 (protein S). Models were adjusted for age, sex, BMI, physical activity, smoking pattern, diabetes mellitus, cancer, estimated glomerular filtration rate, and hs-CRP. Reference points were set to the minimum OR-value

After imputation, multivariable logistic regression analyses revealed associations of the coagulation factors aPTT, protein C, and protein S with VTE (Fig. 1); aPTT (adjusted *p* = 0.016), protein S (adjusted *p* = 0.008), and protein C were non-linearly inversely associated with VTE (adjusted *p* = 0.008). However, there were no associations between prothrombin time, AT, fibrinogen, d-dimers, as well as factor VIII and VTE (Table 3).

**Table 3:** Odds ratio (OR) and 95% confidence interval (95% CI) for the association between blood coagulation factors and VTE as assessed by multivariable logistic regression models

Exposure	OR	95% CI	*p*-value	Adjusted *p*-value^c^
Prothrombin time [%]^a^	0.99	0.95–1.05	0.984	1
aPTT [sec.]^a,^^b^	Non-linear		0.002	0.016
Antithrombin [%]	1.01	0.97–1.05	0.592	1
Fibrinogen [mg/dL]	1.01	1.00–1.01	0.100	0.8
D-dimer [µg/L]	1.00	1.00–1.00	0.599	1
Protein C [%]	Non-linear		0.001	0.008
Protein S [%]	Non-linear		0.001	0.008
Factor VIII [%]	1.01	1.00–1.02	0.092	0.736

Logistic regression models, adjusted for age, sex, BMI, physical activity, smoking pattern, diabetes mellitus, cancer**,** estimated glomerular filtration rate**,** and hs-CRP

^a^Twenty-eight participants with anticoagulation therapy were excluded

^b^Activated partial thromboplastin time

^c^*p*-values were adjusted using the Bonferroni correction

## Discussion

In this population-based cross-sectional study with 805 participants, the prevalence of self-reported VTE was 3.2%. The results of the regression analyses revealed that protein C, protein S, and aPTT were non-linearly inversely associated with VTE.

A VTE prevalence of 3.2% estimated in our population-based study lies within the reported range of 2.9% and 5.1% provided by the health-reporting system in Germany [16].

The results of the present study indicated that plasma fibrinogen and factor VIII concentrations were not associated with VTE prevalence, which is contrary to prior, mostly case–control studies [16–18]. However, the GATE Study [19], a case–control study including 2454 black and white adults showed that factor VIII remained significantly positively associated with VTE in multivariable analysis in black participants only [19]. In their review article, Aleman et al. concluded that plasma fibrinogen concentrations > 4 mg/mL significantly increased the risk of VTE [20], and in the present study, this range was not reached. In addition, a large Danish prospective cohort study, which included over 77,000 individuals from the general population, reported that elevated fibrinogen levels were associated with an increased risk of pulmonary embolism in combination with deep vein thrombosis, but not with DVT alone [21].

In our study, a non-linear association between protein C concentrations and the prevalence of VTE was found. This finding is in accordance with prior research, which consistently demonstrated that low plasma protein C levels, whether due to genetic or acquired causes, are associated with an increased risk of VTE [16, 22]. In the Atherosclerosis Risk in Communities (ARIC) prospective cohort, for example, individuals with protein C levels below 2.0 mg/L (about 1.1% of the population) experienced a 3.36-fold higher incidence of VTE compared to those with higher levels, after adjusting for age. Different genetic mutations causing protein C deficiency (e.g., factor V Leiden) were also shown in several studies to increase the risk for VTE [17, 22–24].

No significant association was found between plasma AT concentrations and VTE in our study, and earlier studies reported mixed results. O. Egeberg was the first to publish findings of a (family-based) genetic study proposing a link between AT deficiency and VTE [25]. A case–control study from Italy reported a twofold increased risk of VTE in patients with low or borderline levels of AT [23]. Furthermore, findings from the population-based MEGA (Multiple Environmental and Genetic Assessment of risk factors for venous thrombosis) cohort study, including patients with first-time VTE, supported the hypothesis that AT deficiency increases the risk of VTE occurrence [26]. However, in the LITE Study, which included participants from ARIC and the Cardiovascular Health Study, no significant association between AT deficiency and VTE was observed [22].

Here, we identified a significant non-linear inverse association between protein S and VTE. The Leiden Thrombophilia Study reported that low levels of protein S could increase the risk for VTE [16]. The population-based MEGA study confirmed these findings, but only for extremely low levels of protein S. The authors pointed out that protein S deficiency is very rarely found in populations; thus, these findings cannot be implemented in clinical practice yet [27]. A case–control study in a Han Chinese population (603 VTE patients, 584 controls) found that individuals with protein S deficiency had a significantly increased risk of VTE after adjusting for age and gender [28]. A cross-sectional study, including data from the UK Biobank and the US National Institutes of Health All of Us, also suggested that protein S deficiency is a rare condition but confers a stronger risk for VTE in the general population than previously thought [29].

A significant non-linear association was observed between aPTT and self-reported VTE in the present investigation. The ARIC Study found that individuals with lower baseline aPTT had a significantly higher risk of developing VTE over 13 years of follow-up, independent of fibrinogen, factors VIII, IX, XI, and von Willebrand factor [30]. A case–control study involving 605 patients with documented VTE and 1,290 controls showed that a shortened aPTT was independently associated with an increased risk of VTE. The authors found these results biologically plausible because faster clotting times are generally associated with higher levels of thrombosis-inducing markers and therefore with a higher risk for VTE [31]. Additionally, a British cohort reported that men with the shortest aPTT levels had a twofold increased risk of VTE after adjusting for age, BMI, smoking, d-dimers, and factor VIII [32].

The biomarkers aPTT, protein C, and protein S play key roles in coagulation and anticoagulation. Mechanistically, a low aPTT is likely to reflect increased levels of coagulation factors, representing an underlying hypercoagulable state [33]. Biologically, protein C and S are vitamin K-dependent glycoproteins. Activated protein C (with protein S as a cofactor) inactivates factors Va and VIIIa, thereby down-regulating thrombin generation and preventing excessive coagulation [34].

Observed associations with VTE may be the consequence of causal prothrombotic pathways (e.g., genetic deficiencies leading to hypercoagulability) or effects of chronic disease (e.g., inflammation or acute-phase responses altering levels post-thrombosis). There is evidence to distinguish causal from non-causal associations [35–38]. While low protein C and S are primarily based on genetic deficiencies and directly prothrombotic, causing VTE via impaired clot inhibition, aPTT and fibrinogen are less clearly causal, acting more as risk markers. They are strongly influenced by inflammation (e.g., in cancer or autoimmune diseases) or chronic conditions, such as obesity or diabetes.

## Study strengths and limitations

All procedures in the KORA-Fit study were standardized, i.e., following standard operating procedures. This refers to the interview, the physical examinations, and the bio-specimen collection, all of which were conducted by trained staff. The information about a physician-based diagnosis of VTE was solely self-reported (by the study participants); it was not validated by confirmation from the treating physician. However, self-reported data may be biased due to a lack of precise knowledge of medical conditions or impaired memory, thus introducing recall bias. In addition, misclassification bias may arise as conditions like PE are often underdiagnosed, leading to false-negative classification of non-detected cases, which impairs the accuracy of the results. As KORA-Fit is a cross-sectional study, coagulation markers and VTE prevalence were assessed contemporaneously (and not prospectively); therefore, no causal conclusions can be drawn from our results. A longitudinal study would overcome parts of this limitation. Also, the lack of clarity on the timing of the testing may have introduced confounding interference, as fibrinogen, factor VIII, and hs-CRP are acute-phase reactants. Furthermore, no information on recent inflammation or hormonal therapy was available; thus, unmeasured confounding cannot be excluded. In addition, the present findings were not validated in another (population-based) study. As this study was conducted in a German population (with a median age of 63 years), the transferability of the results to other populations may be limited.

## Conclusions

Our findings demonstrated significant associations between specific blood coagulation factors and self-reported VTE in the middle-aged and elderly general population. However, due to the cross-sectional study design, self-reported outcome, and small case numbers, the present results should be considered as hypothesis-generating. Further large-scale, prospective studies are required to confirm these associations, evaluate their predictive value in diverse populations, and explore how incorporating coagulation factor measurements might improve clinical outcomes. Further research is needed to deepen our understanding of the complex interaction between coagulation factors and patient-specific risk factors in the pathogenesis of VTE.

## Supplementary Information

Supplementary Material 1.

## Data Availability

The data are subject to national data protection laws, and restrictions were imposed by the Ethics Committee of the Bavarian Chamber of Physicians to ensure data privacy of the study participants. Therefore, data cannot be made freely available in a public repository. However, data can be requested through an individual project agreement with KORA via the online portal KORApasst ([https://epi.helmholtz-muenchen.de/] (https://epi.helmholtz-muenchen.de)).
