# Supplementary material for: Blood coagulation factors and risk of venous thromboembolism: a population-based study
Source: Eur J Med Res. 2026 Jan 11;31:248. doi: 10.1186/s40001-025-03799-3 (PMC12882583; doi:10.1186/s40001-025-03799-3)
Supplement: Supplementary file 1 — Supplementary Material 1. [file 40001_2025_3799_MOESM1_ESM.docx]

**Supplementary Material**

**Blood coagulation factors and risk of venous thromboembolism: a population-based study**

Anna Erhard, Dennis Freuer, Annette Peters, Margit Heier, Christine Meisinger, Jakob Linseisen


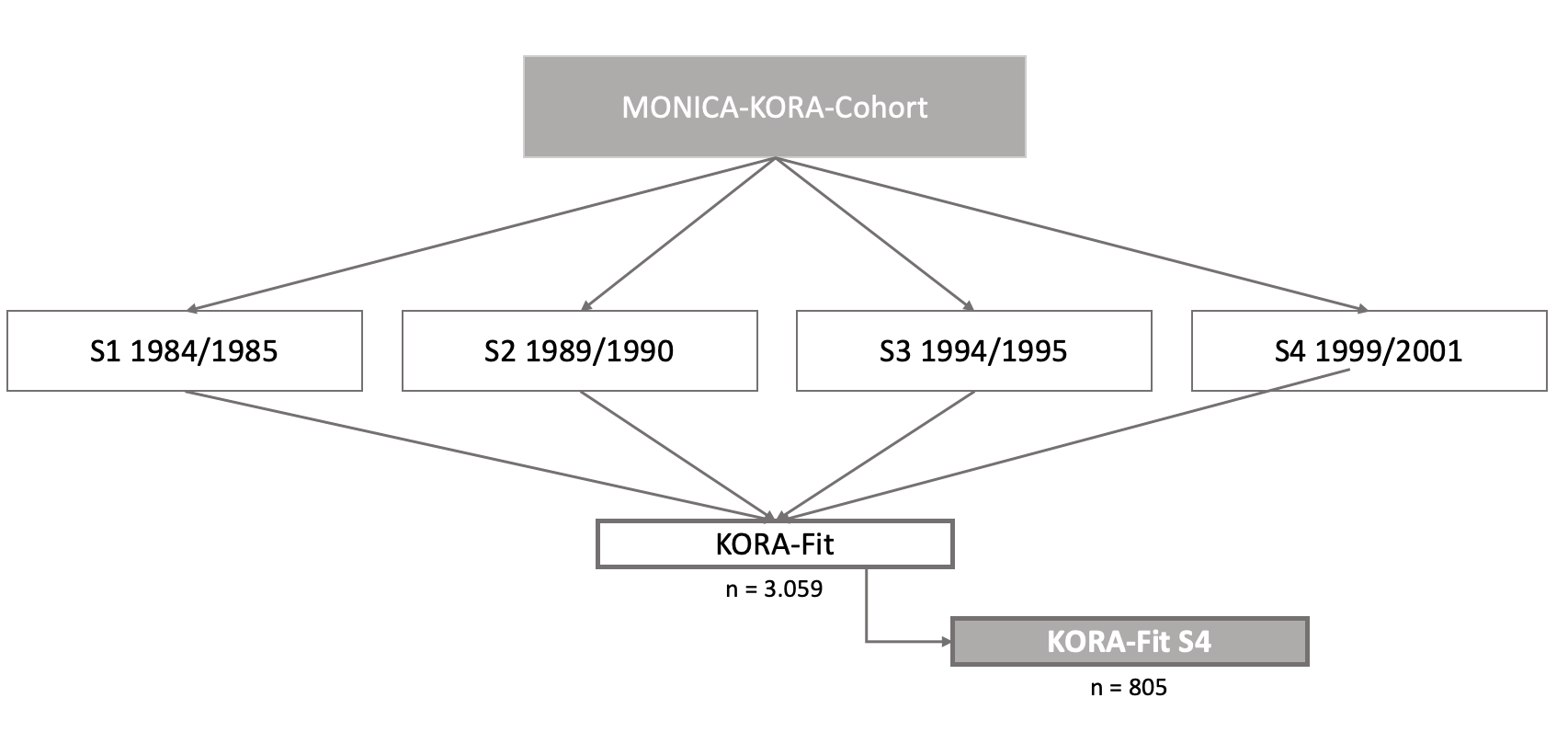


**Figure S1:** The four cross-sectional studies (S1-S4) forming the MONICA-KORA cohort study, and the KORA-Fit examination (2018-2019) inviting participants born between 1945 – 1964, also from the S4 sample (KORA-Fit S4).

**Table S1:** Methods used for the analysis of blood coagulation factors in citrate plasma samples of KORA-Fit S4 participants

| Blood coagulation factor | Reference value | Measurement Technique | Assay or Reagents | Measuring device |
| --- | --- | --- | --- | --- |
| Prothrombin time | 82 – 125 % | Photometric assay | Thromborel S Siemens Eschborn | BCS-XP, Siemens Eschborn |
| aPTT^a^ | 26 – 36 sec. | Photometric assay | Pathromtin SL, CaCl2 solution, Actin FS, Siemens Eschborn | BCS-XP, Siemens Eschborn |
| Antithrombin | 83 – 118 % | Chromogenic assay | Innovance Antithrombin, SCS Cleaner, Siemens Eschborn | BCS-XP, Siemens Eschborn |
| Fibrinogen | 210 – 400 mg/dl | Photometric and turbidimetric assay | Multifibren U, Siemens Eschborn | BCS-XP, Siemens Eschborn |
| D-dimer | < 500 µg/L | Turbidimetric assay | Innovance D-Dimer Kit, Siemens Eschborn | BCS-XP, Siemens Eschborn |
| Protein C | 70 – 140 % | Photometric assay | Berichrom Protein C, Siemens Eschborn | BCS-XP, Siemens Eschborn |
| Protein S | women: 52 – 126 %  men: 73 – 130 % | Photometric assay | Hemoclot Protein S, OVB-Puffer, CaCl2, SCS-Cleaner | CaoChrom, Wien |
| Factor VIII | 70 – 150 % | Photometric assay | Factor VIII deficient plasma, Pathromtin SL, CaCl2, Siemens Eschborn | BCS-XP, Siemens Eschborn |
| ^a^ activated partial thromboplastin time | | | | |

**Table S2:** Calculated AICs regarding non-linear associations for the number of knots between three and five

| **Number of knots** | **AIC for aPTT** | **AIC for Protein C** | **AIC for Protein S** |
| --- | --- | --- | --- |
| 3 | 162.91 | 210.52 | 205.79 |
| 4 | 164.14 | 211.15 | 206.83 |
| 5 | 164.09 | 211.76 | 208.92 |

**Table S3:** P values representing results of testing the non-linearity using restricted cubic splines

|  | **aPTT** | **Prothrombin**  **time** | **Factor VIII** | **Antithrom-bin** | **Fibrino-gen** | **D-dimer** | **Protein C** | **Protein S** |
| --- | --- | --- | --- | --- | --- | --- | --- | --- |
| **age** | 0.505 | 0.577 | 0.972 | 0.968 | 0.581 | 0.999 | 0.953 | 0.880 |
| **BMI** | 0.468 | 0.530 | 0.838 | 0.810 | 0.334 | 0.815 | 0.251 | 0.404 |
| **eGFR** | 0.442 | 0.660 | 0.562 | 0.570 | 0.530 | 0.592 | 0.456 | 0.559 |
| **Exposure** | 0.002 | 0.489 | 0.699 | 0.682 | 0.065 | 0.984 | 0.047 | 0.0003 |
